# Increased Interleukin-23 in Hashimoto’s Thyroiditis Disease Induces Autophagy Suppression and Reactive Oxygen Species Accumulation

**DOI:** 10.3389/fimmu.2018.00096

**Published:** 2018-01-29

**Authors:** Tingting Zheng, Chengcheng Xu, Chaoming Mao, Xiao Mou, Fei Wu, Xuefeng Wang, Ling Bu, Yuepeng Zhou, Xuan Luo, Qingyan Lu, Hongli Liu, Guoyue Yuan, Shengjun Wang, Deyu Chen, Yichuan Xiao

**Affiliations:** ^1^Department of Nuclear Medicine, The Affiliated Hospital of Jiangsu University, Zhenjiang, China; ^2^Institute of Oncology, The Affiliated Hospital of Jiangsu University, Zhenjiang, China; ^3^Department of Laboratory Immunology, Jiangsu University School of Medicine, Zhenjiang, China; ^4^Key Laboratory of Stem Cell Biology, Institute of Health Sciences, Shanghai Institutes for Biological Sciences, Chinese Academy of Sciences and Shanghai Jiao Tong University School of Medicine, Shanghai, China

**Keywords:** autophagy, Hashimoto’s thyroiditis, interleukin-23, reactive oxygen species, AKT/mTOR/NF-κB

## Abstract

Hashimoto’s thyroiditis (HT) represents the most common organ-specific autoimmune disease. Inflammatory factors and reactive oxygen species (ROS) play detrimental roles during the pathogenesis of HT. In this study, we found that thyroid follicular cells (TFCs) from HT patients expressed an elevated level of interleukin-23 (IL-23), which contributed to autophagy suppression and ROS accumulation. Additionally, IL-23-induced autophagy suppression and ROS accumulation in human TFCs was attributed to AKT/mTOR/NF-κB signaling pathway activation. Inhibition of either IL-23 by a specific neutralization antibody, or mTOR by rapamycin, or NF-κB by IKK-16, significantly reversed the autophagy suppression and ROS accumulation. These results demonstrate a key role for IL-23 in HT pathogenesis and provide a potential therapeutic strategy against IL-23 or its signaling pathway in HT.

## Introduction

Hashimoto’s thyroiditis (HT) is one of the most common organ-specific autoimmune diseases and is induced by the loss of immune tolerance for the thyroid gland (1). It is characterized by diffuse infiltration of the thyroid by lymphocytes, destruction of the thyroid gland structure, and autoantibody production (2, 3). However, this needs to be further explored, as HT remains a widespread and multifaceted disease of unknown pathogenesis.

Several studies have shown that reactive oxygen species (ROS) function at physiological concentrations and mediate various biological responses (4, 5). However, excessive ROS generation acts as a more important mechanism for inflammation in virtually all organ systems (6–8). For example, excess reactive oxygen occurs in lymphocytic inflammation disorders, and these disorders are related to atopic dermatitis, inflammation, lupus, and multiple sclerosis (9, 10). Under normal physiological conditions, ROS is essential for thyroid hormone synthesis; however, aberrantly high oxidative stress levels can induce the damage to thyroid follicular cells (TFCs), thyroid gland inflammation, and finally, promote HT development (11, 12). There is evidence showing that thyroid disease prevalence is higher in women because of a higher oxidative stress level in the female thyroid gland (13, 14). However, the mechanism of induction of excess ROS accumulation is not fully understood during HT pathogenesis.

Autophagy is a highly conserved physiological process. Intracellular components undergo lysosome-mediated self-digestion and recycling, and damaged or aged biological macromolecules and organelles are removed from the cytoplasm (15, 16). In quiescent cells, autophagy occurs at a basal level to remove defective organelles, misfolded proteins, and excess protein accumulation in response to endoplasmic reticulum stress (17). Additionally, autophagy is also an important clean-up mechanism of excess ROS in order to protect against damage and death of cells (8, 18). Thus, autophagy is required for the growth, development, function, and survival of normal cells (19–21). A defect in autophagy has been found to confer susceptibility to several autoimmune and inflammatory disorders through the regulation of many critical aspects of disease conditions (7, 21, 22). However, the mechanism of autophagy in autoimmune diseases, such as HT, remains elusive.

Interleukin-23 (IL-23) is a member of the IL-12 superfamily and is composed of an IL-23-specific subunit, IL-23p19 (IL-23a) and a subunit shared with IL-12 (IL-12p40) (23). It has been established that IL-23 is essential for disease development in several models of autoimmune disease, such as psoriatic skin inflammation, experimental autoimmune encephalomyelitis, and rheumatoid arthritis (24–26). It was reported that there is an increased serum concentration of IL-23 in HT patients (27), and IL-23 exerts its pathogenic role in HT development through the promotion of Th17 cell differentiation and IL-17 secretion (28, 29). In this study, we uncovered a novel mechanism that IL-23 contributes to autophagy suppression and ROS accumulation in TFCs during HT pathogenesis.

## Results

### IL-23 and IL-23R Expression Levels Are Enhanced in the Thyroid Tissue of HT Patients and Are Induced in Response to Pro-inflammatory Factors in TFCs

**Figure 1:**
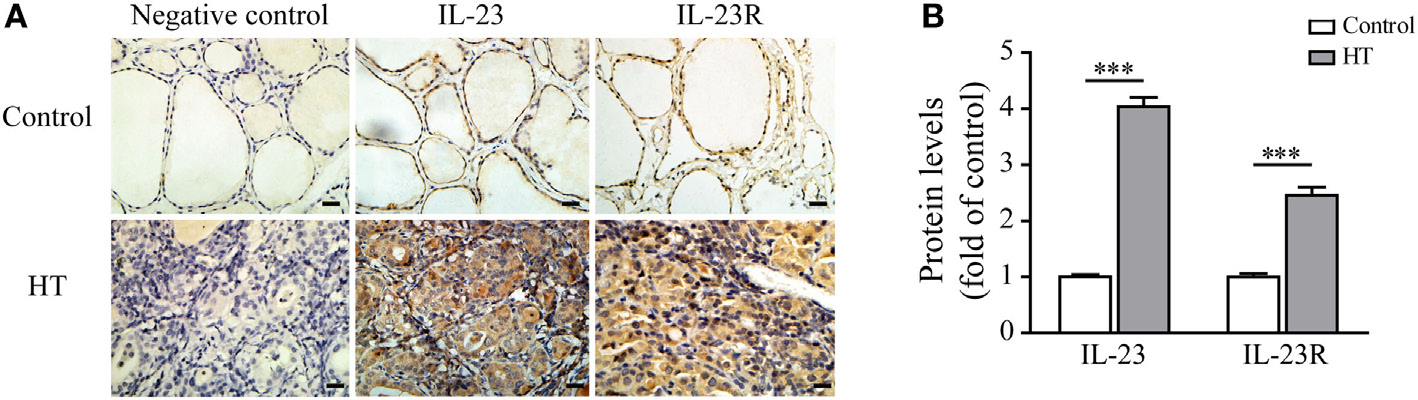
Interleukin-23 (IL-23) and IL-23R expressions in thyroid tissues from Hashimoto’s thyroiditis (HT) patients. **(A)** Thyroid gland sections from HT patients (*n* = 10) and control subjects (*n* = 5) were stained for IL-23 or IL-23R. Representative immunohistochemistry (IHC) results for IL-23 or IL-23R expression are shown. Brown regions represent positive expression of IL-23 or IL-23R. The slides were analyzed under a 400× microscope equipped with a camera (scale bars, 50 µm). **(B)** Cumulative IHC quantification data from 10 HT patients and 5 control subjects are shown. Significant differences and *P* values are calculated with the Mann–Whitney *U* test. ***P < 0.001 vs. controls.

Recent studies have shown that the serum IL-23 concentrations were significantly increased in HT patients in comparison with both Grave’s disease (GD) patients and healthy subjects (30). Therefore, we focused our attention on the expression of IL-23 and its receptor in the thyroid gland of HT patients. The results showed that a low level of IL-23 was expressed in the healthy control TFCs by immunohistochemistry (IHC) examination (*n* = 5; Figure 1). In contrast, IL-23 expression was markedly upregulated in the HT patient TFCs (*n* = 10; Figure 1). Additionally, IL-23R expression in the HT patient TFCs exhibited a similar pattern to the IL-23 expression, and it was also significantly increased compared with the healthy controls (*n* = 5; Figure 1). Cumulative data of the IHC quantification from all samples are shown in Figure 1B. These results suggest that IL-23 expression was enhanced in TFCs under autoimmune inflammatory conditions, and it might be involved in the regional autoimmunity of HT.

**Figure 2:**
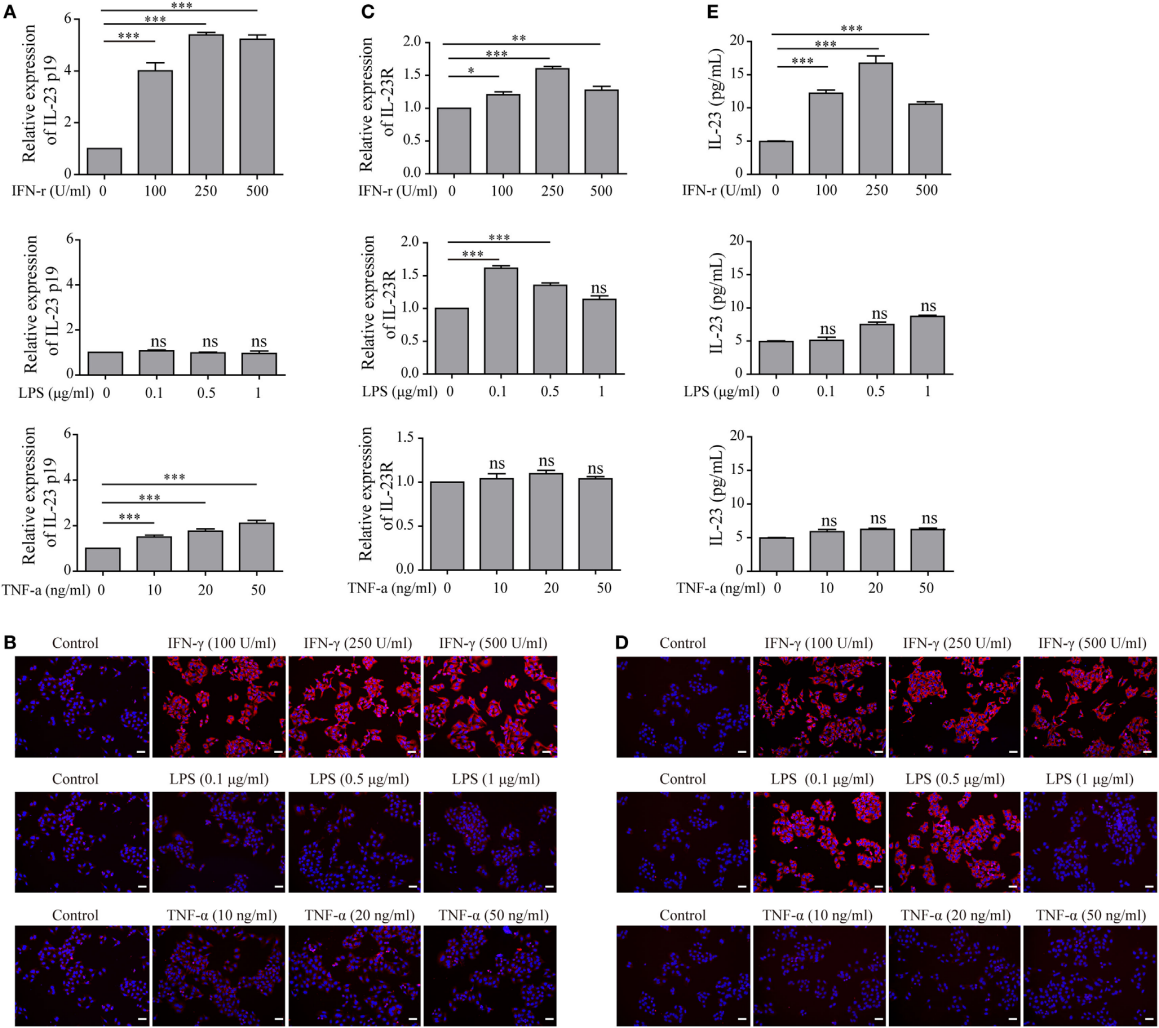
Interleukin-23 (IL-23) and IL-23R expressions in Nthy-ori 3-1 cells in the presence of multiple inflammatory stimuli. Nthy-ori 3-1 cells were treated with the indicated concentrations of interferon-gamma (IFN-γ), lipopolysaccharide (LPS), or tumor necrosis factor alpha (TNF-α). **(A,C)** The IL-23p19 and IL-23R mRNA levels in Nthy-ori 3-1 cells were examined by RT-PCR assay after treatment for 6 h. **(B,D)** Nthy-ori 3-1 cells were treated for 24 h and then stained with anti-IL-23 or anti-IL-23R (red), and their nuclei were counterstained with DAPI (blue). The IL-23 **(B)** or IL-23R **(D)** expression levels were examined by immunofluorescence (200×; scale bars, 50 µm). **(E)** The supernatants were then collected, and the IL-23 concentration was detected by ELISA analysis. The results shown are representative of at least three replicates. Significant differences and *P* values are calculated with the one-way ANOVA. ns, not significant. *P < 0.05, ***P < 0.001 vs. controls.

To determine whether TFC-derived IL-23 was induced under the HT inflammatory conditions, we applied an immortalized human TFC cell line, Nthy-ori 3-1 cells, for further experiments. The mRNA expression of IL-23p19 and IL-23R in Nthy-ori 3-1 cells were examined with an RT-PCR assay in the presence of interferon-gamma (IFN-γ), lipopolysaccharide (LPS), and tumor necrosis factor alpha (TNF-α), respectively. The results showed that the IL-23p19 expression in Nthy-ori 3-1 cells was sharply increased by IFN-γ stimulation, but not by LPS or TNF-α, compared with the control group (Figure 2A). Equally, the IL-23 protein expression levels exhibited a similar pattern to the IL-23p19 mRNA expression levels, as shown by an immunofluorescence assay (Figure 2B). Additionally, the expression of the IL-23-specific receptor, IL-23R, both in the level of mRNA and on the surface of the Nthy-ori 3-1 cells was significantly induced after treatment with IFN-γ or LPS, but not TNF-α, compared with the control (Figures 2C,D). We also found that the level of IL-23 in the culture supernatants was increased after treatment with IFN-γ compared with the control. LPS stimulation also showed a slight increase in IL-23 secretion from TFCs (Figure 2E). These results collectively suggest that the pro-inflammatory cytokine, IFN-γ, is a major inducer of TFC-derived IL-23.

### IL-23 Induces ROS Accumulation and Autophagy Inhibition in TFCs

**Figure 3:**
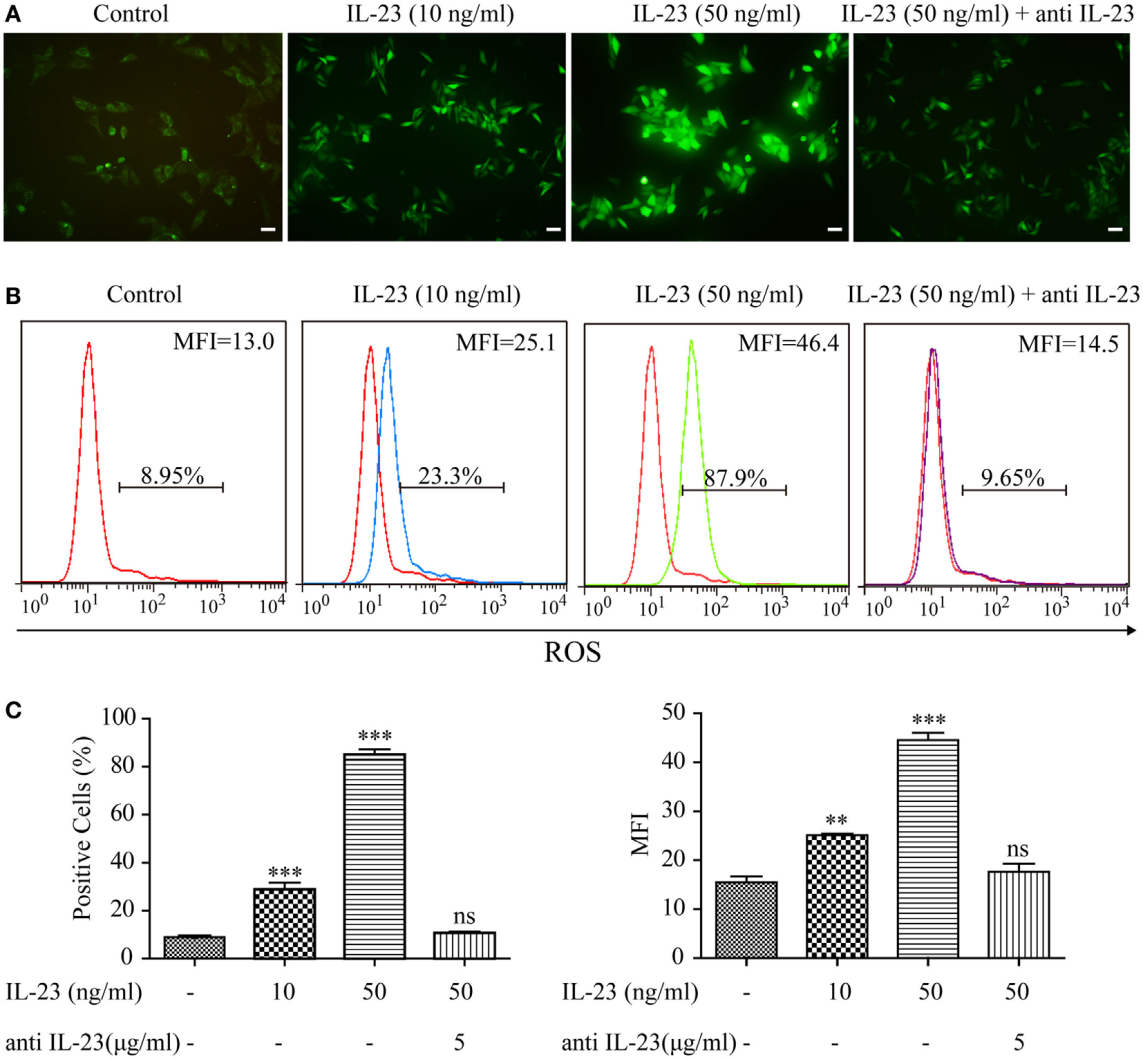
Effect of interleukin-23 (IL-23) on intracellular reactive oxygen species (ROS) levels in Nthy-ori 3-1 cells. Nthy-ori 3-1 cells were incubated with the indicated concentrations of IL-23 for 3 h in the presence or absence of anti-IL-23 (5 µg/mL) pre-treatment, and the cellular ROS was probed for with DCFH-DA. **(A)** The DCF fluorescence was detected and analyzed immediately using immunofluorescence (200×; scale bars, 50 µm). **(B,C)** The intracellular ROS levels by flow cytometry analysis are presented by percentages of positive cells (% positive cells) and mean fluorescence intensity (MFI). The statistical results shown are representative of three replicates. Significant differences and *P* values are calculated with the one-way ANOVA. ns, not significant. **P < 0.01, ***P < 0.001.

As aberrant ROS accumulation is a detrimental mediator for HT (13), to determine whether the increased IL-23 expression in TFCs indeed contributes to HT pathogenesis, we utilized Nthy-ori 3-1 cells to examine the effect of IL-23 on intracellular ROS levels. After treatment with varying IL-23 concentrations for 3 h, Nthy-ori 3-1 cells were stained with DCFH-DA, and ROS accumulation was detected by both immunofluorescence and flow cytometric methods. The results showed that the ROS production in Nthy-ori 3-1 cells was induced by IL-23 in a dose-dependent manner (Figure 3). To directly confirm that the ROS regulation was dependent on the IL-23-mediated signaling pathway, a neutralizing antibody against IL-23 was pretreated in the experiment system. The results showed that antiIL-23 antibody treatment abolished the IL-23-induced ROS accumulation in Nthy-ori 3-1 cells (Figure 3). These findings suggest that IL-23 mediated the ROS accumulation in TFCs.

**Figure 4:**
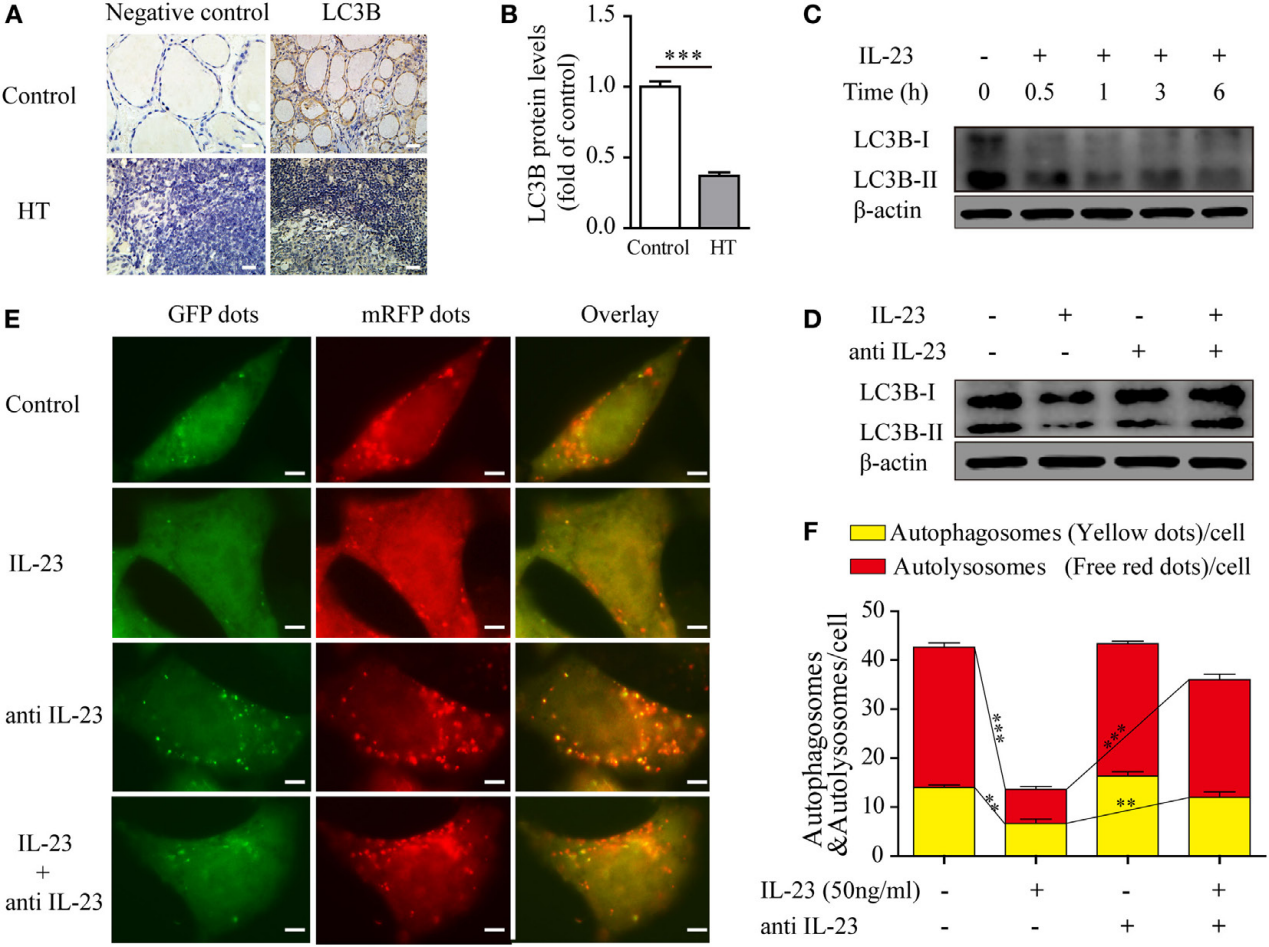
Expression of the autophagy-related protein LC3B in thyroid tissues of Hashimoto’s thyroiditis (HT) patients and the effect of interleukin-23 (IL-23) on autophagy activity in Nthy-ori 3-1 cells. **(A)** The levels of the autophagy-related protein LC3B in thyroid tissues from HT patients (*n* = 10) and healthy controls (*n* = 5) were examined with immunohistochemistry (IHC) analysis. The brown regions represent positive expression. The slides were analyzed under a 400× microscope equipped with a camera (scale bars, 50 µm). **(B)** Cumulative IHC quantification data from all samples are shown. Significant differences and *P* values were calculated with the Mann–Whitney *U* test. ***P < 0.001 vs. controls. **(C)** Nthy-ori 3-1 cells were treated with IL-23 (50 ng/mL) at different time points, the LC3B-I/II expression levels were measured by western blot. **(D)** The change of LC3B-II expression in Nthy-ori 3-1 cells was analyzed at 3 h by western blot in the presence of IL-23 (50 ng/mL) and/or anti-IL-23 pretreatment (5 µg/mL). **(E)** Nthy-ori 3-1 cells were transfected with mRFP-GFP-LC3, and then were treated with IL-23 (50 ng/mL) and/or anti-IL-23 pre-treatment (5 µg/mL). Representative images of the fluorescent LC3 puncta are shown (1,000×; scale bars, 5 µm). **(F)** Mean number of autophagosomes (puncta with yellow color in the merged images) and autolysosomes (puncta with only red color in the merged images) per cell were analyzed. The results shown are representative of three replicates. Significant differences and *P* values are calculated with the Kruskal–Wallis *H* test. **P < 0.01, ***P < 0.001.

Under normal physiological circumstances, autophagy prevents mitochondrial ROS release by digesting dysfunctional mitochondria, which is known to activate the inflammasome (6). Therefore, we speculated that IL-23-induced ROS accumulation in TFCs may be due to autophagy inhibition. To test this hypothesis, we first measured autophagy activity in thyroid tissue from HT patients using an IHC technique. The level of the autophagy-related protein, light chain 3 (LC3B), which is incorporated into autophagosomes and serves as a well-characterized indicator of autophagy activation, was highly expressed in the healthy control TFCs. However, LC3B protein expression in TFCs was sharply decreased in HT tissues compared with the healthy controls (Figures 4A,B). Considering that the IL-23/IL-23R expression levels were significantly increased in the HT patient TFCs, we performed *in vitro* experiments to test whether IL-23 contributed to autophagy inhibition. Nthy-ori 3-1 cells were cultured in the presence of IL-23 (50 ng/ml) for different time points, and LC3B-I/II expressions were analyzed by western blot. As expected, the IL-23 treatment significantly inhibited LC3B-II expression in Nthy-ori 3-1 cells (Figure 4C). Furthermore, the IL-23-induced inhibition of the autophagy-related protein, LC3B-II, was reversed by the addition of a neutralizing antibody against IL-23 in Nthy-ori 3-1 cells (Figure 4D).

To further confirm the inhibitory effect of IL-23 in autophagy, we applied an autophagy tandem reporter, mRFP-GFP-LC3, to infect Nthy-ori 3-1 cells, and the autophagic flux status was measured. The lysosomal environment can quench GFP fluorescence but does not affect mRFP fluorescence; therefore, the autophagosome appears as a yellow dot (the result of merged GFP and mRFP fluorescence) in the cytoplasm, and the autolysosome appears as a red dot in the lysosome (31, 32). As shown in Figures 4E,F, IL-23 treatment significantly reduced both the yellow dots that represent autophagosomes (*P* < 0.01) and the red dots that represent autolysosomes (*P* < 0.001). However, when the neutralizing antibody against IL-23 was added in the culture medium, the fluorescent puncta formation was significantly reversed in the TFCs. Taken together, these findings indicate that IL-23 inhibited autophagy activity, which might contribute to the ROS accumulation in the TFCs of HT patients.

### IL-23-Induced AKT/mTOR/NF-κB Signaling Contributes to Autophagy Suppression and ROS Accumulation

**Figure 5:**
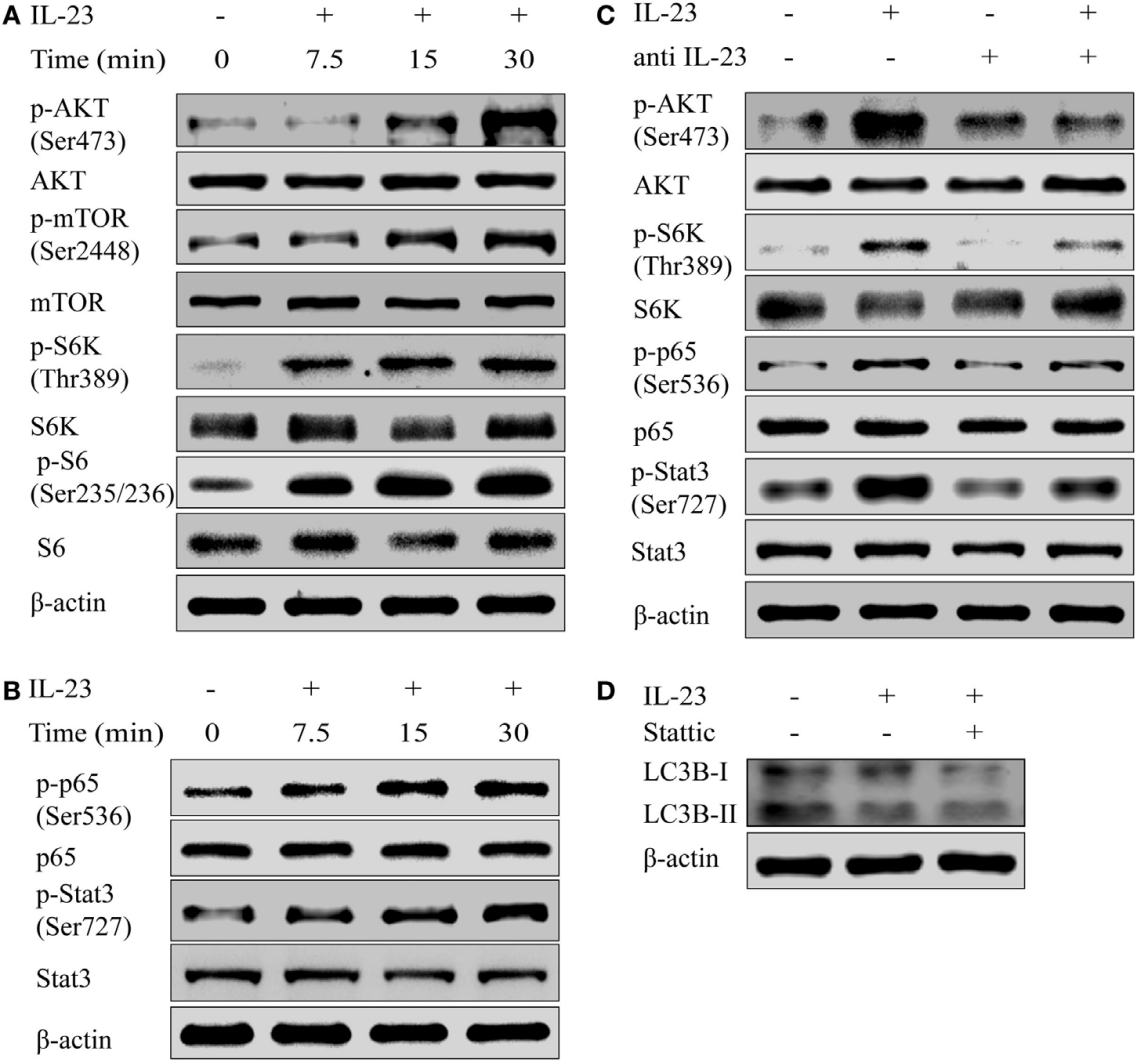
Interleukin-23 (IL-23) induces AKT/mTOR/NF-κB signaling pathway activation. Nthy-ori 3-1 cells were treated with IL-23 (50 ng/mL) at different time points, and changes of AKT, p-AKT (Ser473), mTOR, p-mTOR (Ser2448), S6k, p-S6k (Thr389), S6, and p-S6 (Ser235/236) expression levels **(A)**, as well as NF-κB p65, p-NF-κB p65 (Ser536), Stat3, and p-Stat3 (Ser727) expression levels **(B)**, were determined by western blot. **(C)** Nthy-ori 3-1 cells were treated with IL-23 (50 ng/mL) and/or anti-IL-23 pre-treatment (5 µg/mL), changes of AKT, p-AKT (Ser473), S6k, p-S6k (Thr389), NF-κB p65, p-NF-κB p65 (Ser536), Stat3, and p-Stat3 (Ser727) expression levels were analyzed by western blot. **(D)** The change of LC3B-II expression in Nthy-ori 3-1 cells was analyzed at 3 h by western blot in the presence of IL-23 (50 ng/mL) with or without Stattic (10 µM) treatment. The results shown are representative of three replicates.

To determine the molecular mechanism by which IL-23 inhibited the autophagy activity, we tested the activation of AKT/mTOR pathway, which is reported to contribute to the autophagosome formation (33). The results showed that IL-23 was a strong inducer of AKT/mTOR signaling pathway activation, exhibiting significantly increased phosphorylation of Akt (Ser473), mTOR (Ser2448), S6K (Thr389), and S6 (Ser235/236) in Nthy-ori 3-1 cells (Figure 5A). Apart from AKT/mTOR activation, IL-23 also promoted NF-κB p65 (Ser536) phosphorylation (an indicator of NF-κB activation), and Stat3 (Ser727) activation (a well-characterized responder to IL-23 stimulation) (Figure 5B). Additionally, neutralizing antibody treatment against IL-23 abolished the IL-23-induced AKT, S6k, p65, and Stat3 (Figure 5C) activation in Nthy-ori 3-1 cells. But the addition of Stattic, a pharmacological inhibitor of Stat3 (Ser727), did not rescue IL-23-induced suppression of LC3B-II protein (Figure 5D).

**Figure 6:**
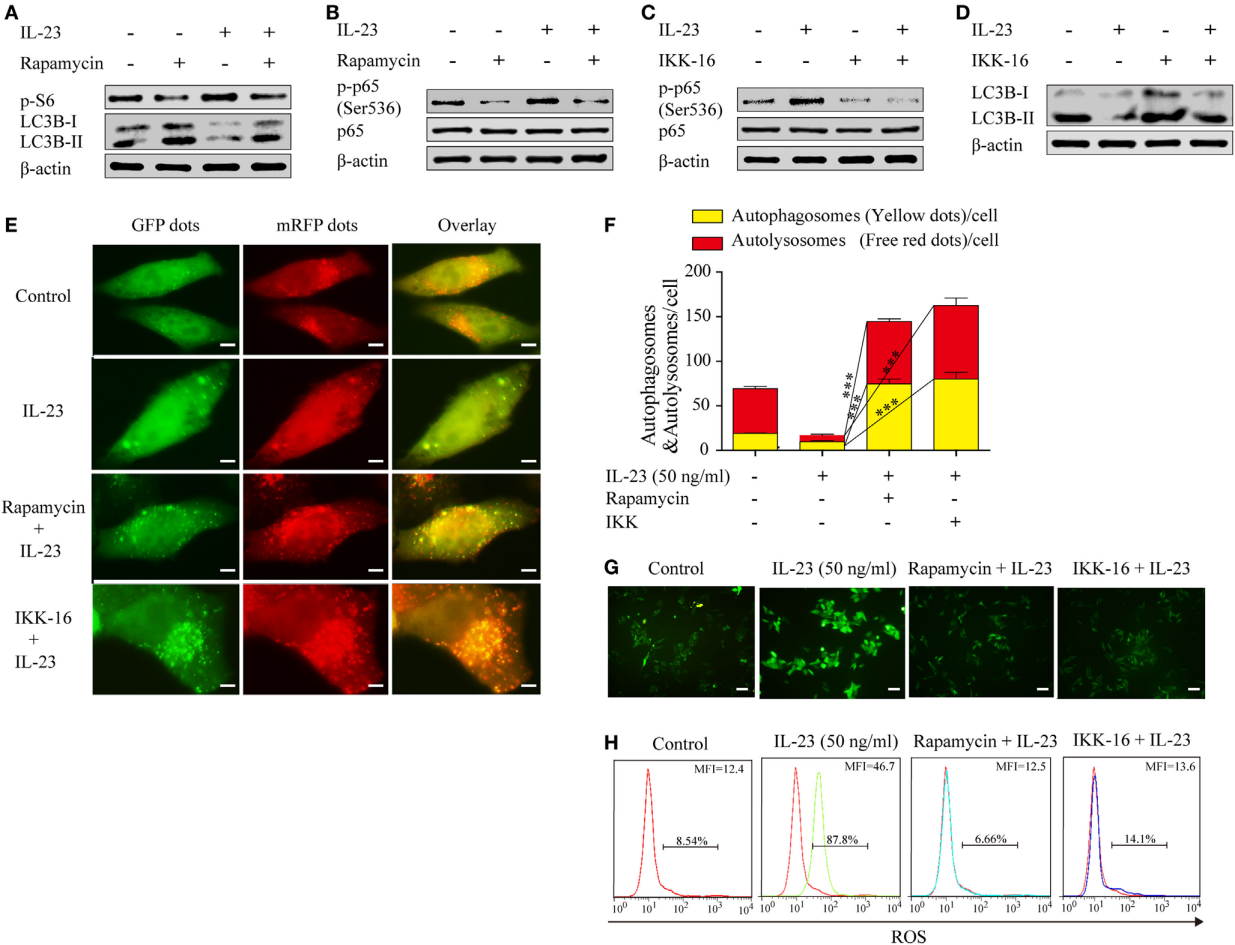
Rapamycin and IKK-16 protect against interleukin-23 (IL-23)-induced autophagy inhibition and reduce IL-23-induced reactive oxygen species (ROS) accumulation. Changes of p-S6 (Ser235/236) and LC3B-II **(A)** as well as NF-κB p65 and p-NF-κB p65 (Ser536) **(B)** expression levels were analyzed by western blot in the presence of IL-23 (50 ng/mL) and/or rapamycin (10 nM). Changes of NF-κB p65 and p-NF-κB p65 (Ser536) **(C)** as well as LC3B-II **(D)** expression levels were detected by western blot in the presence of IL-23 (50 ng/mL) and/or IKK-16 (5 µM). **(E)** Nthy-ori 3-1 cells were transfected with mRFP-GFP-LC3, and then were treated with IL-23 (50 ng/mL) in the presence or absence of rapamycin (10 nM) or IKK-16 (5 µM). Representative fluorescent LC3 puncta images are shown (1,000×; scale bars, 5 µm). **(F)** Mean autophagosome (puncta with yellow color in merged images) and autolysosome (puncta with only red color in merged images) numbers per cell were analyzed. **(G,H)** Nthy-ori 3-1 cells were treated with IL-23 (50 ng/mL) in the presence or absence of Rap (10 nM) or IKK-16 (5 µM), and cellular ROS was probed for with DCFH-DA. The DCF fluorescence was detected immediately using immunofluorescence **(G)** (200×; scale bars, 50 µm) or flow cytometry **(H)**. And flow cytometry are presented by percentages of positive cells (% positive cells) and mean fluorescence intensity (MFI). The results shown are representative of three replicates. Significant differences and *P* values are calculated with the Kruskal–Wallis *H* test. ***P < 0.001.

To determine whether the IL-23-induced AKT/mTOR activation contributes to autophagy inhibition, we took advantage of rapamycin, a pharmacological inhibitor of mTOR that positively regulates autophagy, to examine the autophagy activation. Nthy-ori 3-1 were pretreated with or without rapamycin and then stimulated with IL-23, and the phospho-S6 and LC3B-II expression levels were detected by western blot. A significant decrease in the phosphorylated S6 level in the presence of rapamycin was observed, which was accompanied with increased LC3B-II protein levels (Figure 6A), suggesting that IL-23-induced mTOR activation suppressed autophagy activation.

It was reported that mTOR can regulate NF-κB activity in cancer cells (34), therefore, we sought to determine whether IL-23-induced mTOR is the upstream signaling to regulate NF-κB activation in TFCs. The rapamycin treatment significantly decreased the level of phospho-NF-κB p65 protein in the IL-23-treated cells (Figure 6B), suggesting that mTOR functioned as an upstream regulator to mediate NF-κB p65 activation in IL-23-stimulated TFCs. Additionally, IKK-16, a pharmacological inhibitor of NF-κB, significantly rescued IL-23-induced suppression of the LC3B-II protein (Figures 6C,D). To further confirm the involvement of mTOR and NF-κB in autophagy, we used an autophagy tandem reporter, mRFP-GFP-LC3, to infect Nthy-ori 3-1 cells, and the autophagic flux status was analyzed. The autophagosome (yellow puncta) and autolysosome (red puncta) numbers in the cells were significantly increased after mTOR (rapamycin) or NF-κB (IKK-16) inhibitors treatments in the presence of IL-23 (Figures 6E,F; *P* < 0.001). These findings suggest that IL-23-induced autophagy inhibition is attributed to the activation of AKT/mTOR/NF-κB signaling pathway activation.

To further confirm that the IL-23-induced AKT/mTOR/NF-κB signaling pathway contributes to ROS accumulation in TFCs, the extent of ROS accumulation was examined in Nthy-ori 3-1 cells that were treated with IL-23 in the presence or absence of rapamycin or IKK-16. Expectedly, we found that the addition of rapamycin or IKK-16 both significantly inhibited IL-23-induced ROS accumulation in TFCs (Figures 6G,H). Collectively, we confirmed that IL-23-mediated mTOR/NF-κB activation contributed to autophagy inhibition and ROS accumulation in human TFCs.

### AKT/mTOR and NF-κB Signaling Pathways Are Highly Activated in TFCs during HT Pathogenesis

**Figure 7:**
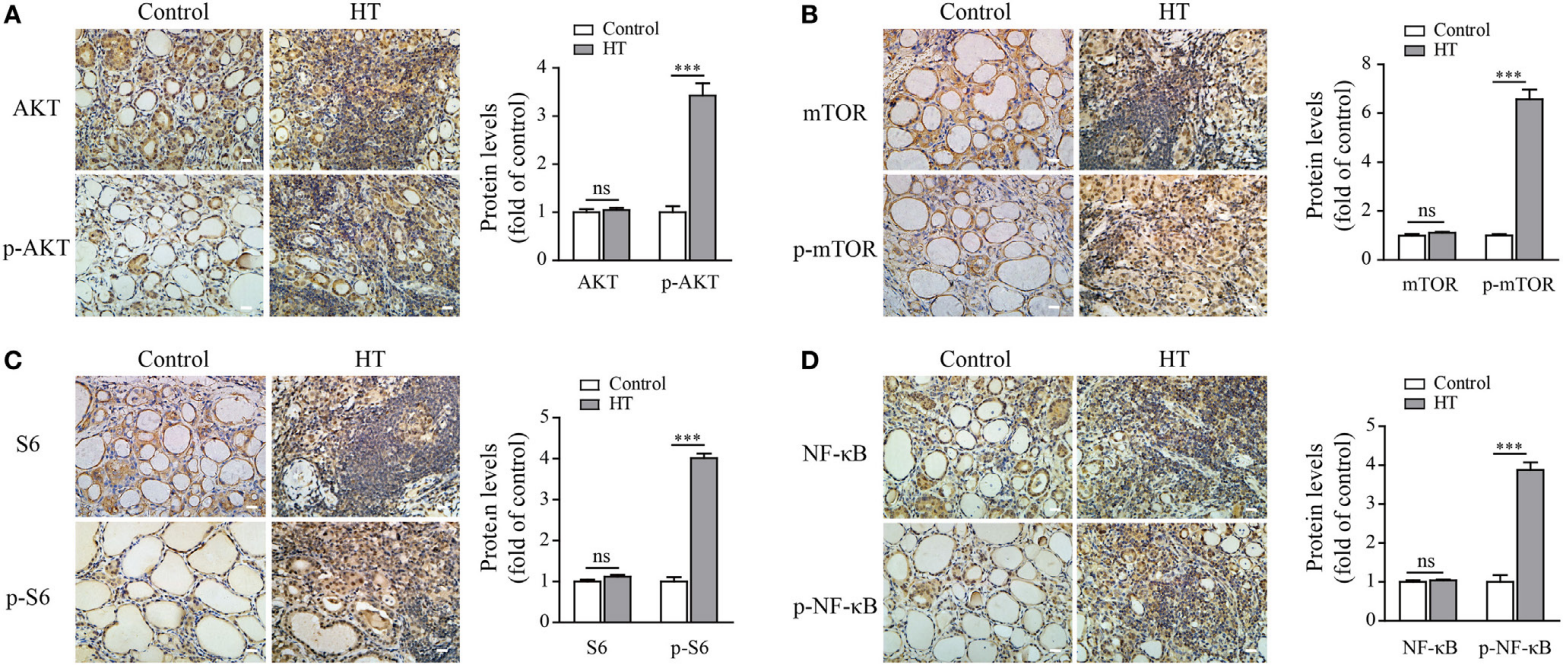
AKT, p-AKT, mTOR, p-mTOR, S6, p-S6, NF-κB p65, and p-NF-κB p65 expression levels in Hashimoto’s thyroiditis (HT) patient thyroid glands. Representative results from HT patients (*n* = 10) and control tissues (*n* = 5) by immunohistochemistry (IHC) staining for AKT and p-AKT **(A)**, mTOR and p-mTOR **(B)**, S6 and p-S6 **(C)**, and NF-κB and p-NF-κB **(D)** are shown (scale bars, 50 µm). The brown regions represent positive expression. The slides were analyzed under a 400× microscope equipped with a camera. The IHC quantification results from all samples are shown in the right panel. Significant differences and *P* values are calculated with the Mann–Whitney *U* test. ns, not significant. ***P < 0.001 vs. controls.

To further confirm that autophagy inhibition and ROS accumulation in TFCs of HT patients were due to the activation of the AKT/mTOR/NF-κB signaling pathway, we evaluated the activation status of these signaling proteins in HT patient tissues by IHC. The results showed that the phosphorylated AKT, mTOR, and its downstream target S6 expression levels were significantly upregulated in the TFCs of HT patient tissues compared with the healthy controls, whereas the total AKT, mTOR, and S6 expression levels in the TFCs were at the similar levels between the healthy controls and HT patients (Figures 7A–C). Additionally, the phosphorylated NF-κB p65 levels were also increased in the TFCs of HT tissues compared with the healthy controls (Figure 7D). These data suggest that the AKT/mTOR/NF-κB signaling pathway was activated in the thyroid tissue of HT patients, which exhibited similar results as the IL-23-induced TFCs *in vitro*.

## Discussion

Hashimoto’s thyroiditis, as one of the most common autoimmune endocrine diseases, is a chronic inflammation disorder of the thyroid gland, which was initially described over a century ago (35). However, the mechanisms that trigger the autoimmune attack to the thyroid gland are still unclear. IL-23 has an important role in the pathogenesis of several inflammatory and autoimmune conditions, mostly through inducing Th17 cell differentiation (23, 36–38). In the present study, we found that IL-23 was highly expressed in the TFCs of HT patients and could be induced by the Th1 signature cytokines, such as IFN-γ, in a human TFC cell line. This discovery is in accordance with the previous reports that a significantly higher serum concentration of IL-23 was observed in HT patients (30). We also found that IL-23R expression in TFCs was induced under inflammatory conditions. Collectively, these results suggested that IL-23 was induced in TFCs under the inflammatory conditions during HT pathogenesis, and might function as an autocrine pattern to stimulate TFCs, which contributed to HT pathology.

It is well-known that ROS are produced by all cells of living organisms and are able to act in the redox-dependent regulation of different cellular functions, including the response to stressors, angiogenesis, and cell proliferation (4). An appropriate amount of ROS in the thyroid gland is necessary for thyroid hormonogenesis. However, excess amounts of ROS contribute to the pathological damage of TFCs and the development of autoimmune thyroid diseases, such as HT and GD (11, 13). The production of ROS and oxidative stress are interlinked with production and activation of various growth factors and cytokines (39, 40). Interestingly, we discovered that IL-23 induced a dramatic increase of ROS accumulation in TFCs, speculating that IL-23-induced ROS accumulation, as an important pathogenic mechanism, participated in the inflammatory process of HT.

Under normal physiological condition, basal autophagy is responsible for clearing the cytoplasm of non-functional mitochondria, protein accumulation, and other organelles. Cell autophagic defect leads to an accumulation of depolarized mitochondria and proteins, which induce the release of inflammasome activators, such as ROS or mitochondrial DNA (6, 19). Autophagy inhibition allows for the processing and secretion of the pro-inflammatory cytokines, and this process may be dependent on ROS accumulation (7, 41). Based on this point, we speculated that IL-23-mediated ROS accumulation is due to an autophagic defect in TFCs. Our data demonstrated that autophagy activation is indeed defective not only in IL-23-stimulated TFCs but also in HT patient thyroid gland tissues. Additionally, the mTOR inhibitor, rapamycin, which is also a commonly used autophagy activator, abolished IL-23-mediated ROS accumulation. Based on these results, we demonstrated that excessive IL-23 in thyroid tissue can suppress autophagy activity, leading to the accumulation of excessive ROS. Furthermore, the excessive ROS promoted inflammation and the production of pro-inflammatory cytokines, including IL-23. The consequence is to form a positive feedback loop and exacerbated the disease severity.

The AKT/mTOR signaling pathway has been reported to play important roles in the regulation of autophagosome formation. Interestingly, we found that IL-23 could activate the AKT/mTOR signaling pathway. Additionally, mTOR inhibition could reverse IL-23-mediated biological functions, including rescuing autophagy inhibition and abolishing ROS accumulation in TFCs. Furthermore, AKT/mTOR activation in TFCs was also found to be highly induced in HT tissues, providing clinical evidence of IL-23-induced AKT/mTOR activation and autophagy suppression. We also presented data that IL-23 could induce NF-κB and STAT3 signaling pathway activation; therefore, it is reasonable that NF-κB and STAT3 activation are involved in the IL-23-induced autophagy suppression and ROS accumulation processes. We found that a STAT3 inhibitor could not rescue the autophagy suppression in TFCs, suggesting that the IL-23-induced STAT3 activation was not involved in this process. However, an NF-κB inhibitor rescued the autophagy suppression and inhibited ROS accumulation in TFCs. Additionally, the mTOR inhibitor, rapamycin, also inhibited NF-κB p65 activation, suggesting that AKT/mTOR may function an upstream regulator of IL-23-induced NF-κB activation in TFCs. Collectively, IL-23-induced autophagy suppression and ROS accumulation in TFCs were dependent on AKT/mTOR/NF-κB signaling pathway activation. Taken together, we speculate that during HT pathogenesis, inflammatory cytokines induce IL-23 production from TFCs, which functions as an autocrine cytokine and inhibits autophagy activation through AKT/mTOR/NF-κB signaling pathway activation. This leads to ROS accumulation in TFCs and HT pathology exacerbation. Therefore, targeting IL-23, autophagy activation or mTOR could provide a potential therapeutic strategy aimed at preventing cellular oxidative damage to thyroid cells.

## Materials and Methods

### Cell Lines and Samples

Nthy-ori 3-1, a human thyroid follicular epithelial cell line from the European Collection of Animal Cell Cultures, was cultivated in RPMI-1640 (Gibco, USA) supplemented with 10% fetal bovine serum (Gibco) in the presence of 5% CO_2_ and 37°C. The patients in this study were recruited from the Hospital Affiliated of Jiangsu University. Thyroid glands were obtained from 10 patients with HT (10 females, aged 34–61; mean age 48.1 ± 2.9 years) who underwent a thyroidectomy. An HT diagnosis was made based on clinical evaluations and Japanese guidelines as described previously (3, 42). All patients were not taking any anti-cytokine therapy and other drugs affecting thyroid function at the time of sampling. Thyroid tissues from five patients with a simple goiter (five females, aged 38–59; mean age 50.6 ± 3.8 years) were used as controls based on clinical evaluations and laboratory findings. All samples were taken in accordance with the regulations and approval of the Institutional Review Board of the Affiliated Hospital of Jiangsu University. In all cases, written informed consent was obtained, and the study was approved by the Ethics Committee of the Affiliated Hospital of Jiangsu University and conducted in accordance with the Declaration of Helsinki guidelines.

### Reagents and Antibodies

Primary antibodies, such as rabbit anti-LC3B; rabbit anti-AKT, rabbit anti-p-AKT (Ser473), rabbit anti-mTOR, rabbit anti-p-mTOR (Ser2448), rabbit anti-p70S6K, rabbit anti-p-p70S6K (Thr389), mouse anti-S6, rabbit anti-p-S6 (Ser235/236), rabbit anti-NF-κB p65, rabbit anti-p-NF-κB p65 (Ser536), rabbit anti-Stat3, rabbit anti-p-Stat3 (Ser727), and rabbit anti-β-actin were obtained from Cell Signaling Technology (USA). HRP-labeled goat anti-rabbit IgG, HRP-labeled goat anti-mouse IgG, PE-labeled goat anti-rabbit IgG secondary antibodies, anti-IL-23, and anti-IL-23R were from Santa Cruz (USA). IFN-γ and TNF-α were from PeproTech (USA). Recombinant human IL-23 protein and human IL-23p19 antibody, as well as the IL-23 Quantikine ELISA kit were from R&D systems (USA). The ROS assay kit was from Beyotime Biotech (China). Rapamycin and LPS were purchased from Sigma-Aldrich (USA). IL-23R was from Abcam. Stattic was from Merck Millipore (USA), and the IKK-16 was from Selleck Chemicals (USA).

### IHC Staining

Thyroid tissues were obtained from the Department of Pathology of The Hospital Affiliated to Jiangsu University. Tissue sections were performed as follows: the samples were fixed in 10% neutralized formalin, embedded in paraffin, sectioned into 4-µm sections, and mounted on slides. After deparaffinization and rehydration, antigen retrieval was performed by boiling the samples in 10 mmol/L citrate buffer (pH 6.0) for 10 min, and the slides were washed with phosphate-buffered saline, blocked with 5% normal goat serum, and then incubated with a primary antibody overnight at 4°C. After three washes with PBS, the sections were treated with the corresponding streptavidin peroxidase-conjugated secondary antibody (Maixin Biotechnology Co., Ltd.). Tissue sections were then counterstained with 3,3′-diaminobenzidine and hematoxylin and observed under a light microscope. The results of quantitative analyses of all samples with the Image-Pro plus 6.0 software (Version X; Media Cybernetics, USA) are presented graphically.

### RT-PCR

Total RNA was extracted from Nthy-ori 3-1 cells with Trizol reagent (Invitrogen, 15596026, USA) according to the manufacturer’s instructions after treatments with IFN-γ, LPS, or TNF-α for 6 h. RNA was eluted with RNase-free water, and the RNA concentrations were determined using a BioMate 3S analyzer (Thermo Fisher Scientific, USA). RT-PCR was performed using the RNA PCR Kit ver. 3.0 (Takara Biotechnology, RR019B, China). Using random hexamers and oligo dT as a primer, 1 µg of total RNA was reverse transcribed in a 20-µl volume with the PrimeScript^®^ RT reagent Kit (Perfect Real Time). The qRT-PCR was performed with SYBR^®^ Premix Ex Taq™ (Tli RNaseH Plus) in a real-time PCR Mx3000PTM System (Genetimes Technology, China). For mRNA detection, the following primers were utilized: IL-23p19 (forward: 5′-cccaaggactcagggacaac-3′; reverse: 5′-agagaaggctcccctgtgaa-3′); GAPDH (forward: 5′-aggtgaaggtcggagtcaac-3′; reverse: 5′-gggtggaatcatattggaaca-3′). GAPDH was used as the internal control. The 2^−ΔΔCt^ method was used to calculate the relative gene expression levels.

### ELISA

Supernatant IL-23 levels were measured by a quantitative enzyme immunoassay technique with the IL-23 Quantikine ELISA Kit according to the manufacture’s instructions (R&D System) after treatments with IFN-γ, LPS, or TNF-α for 24 h. A microplate reader (Thermo Fisher Scientific) capable of measuring absorbances at 450 and 570 nm was used to measure the color intensity that developed in each well. All assays were done in duplicate. The detection limit of the assay was 2.7 pg/mL.

### Intracellular ROS Measurement

The intracellular ROS level was monitored using a fluorescent probe, 2,7-dichlorohydrofluorescein diacetate (DCFH-DA) (Beyotime Biotechnology, China), which is a specific probe for hydrogen peroxide. Briefly, cells were seeded at 2 × 10^5^ cells/well in a six-well plate overnight and treated with indicated concentrations of IL-23 in the presence or absence of anti-IL-23 (5 µg/mL), rapamycin (10 nM), or IKK-16 (5 µM) pre-treatment, followed by incubation with a final concentration of 10 µM DCFH-DA for 30 min at 37°C. The cells were then collected, washed, and resuspended three times in RPMI-1640 without FBS. The DCF fluorescence of 1 × 10^5^ cells was detected and analyzed immediately using flow cytometry (FACS Calibur; BD Biosciences, USA) with an excitation wavelength of 488 nm and an emission wavelength of 525 nm. The intracellular ROS levels by flow cytometry analysis are presented by percentages of positive cells (% positive cells) and mean fluorescence intensity. Additionally, the DCF fluorescence of the ROS production from the Nthy-ori 3-1 cells was assessed using a fluorescence microscope (Olympus, Japan).

### Immunofluorescence Analysis for IL-23 or IL-23R

Nthy-ori 3-1 cells were cultivated in 24-well plates and treated with IFN-γ, LPS, or TNF-α for 24 h to test for IL-23 or IL-23R. First, the plates were washed twice with PBS, fixed in 4% paraformaldehyde at room temperature for 20 min, and permeabilized, and then non-specific receptors were blocked with 0.1% Triton-X-100 and 5% BSA in PBS at room temperature for 30 min. Next, the cells were incubated with primary antibody to detect IL-23 and IL-23R overnight at 4°C. They were then incubated with a secondary PE-labeled antibody at room temperature for 1 h. In all samples, DAPI (Beyotime Biotechnology) was added to visualize DNA in the cell nucleus. These stained cells were observed with a fluorescence microscope.

### Autophagy Determination

Nthy-ori 3-1 cells were infected with adenoviral-expressing mRFP-GFP-LC3 (Hanbio Biotechnology, China) at a multiplicity of infection of 200 for approximately 2 h. To quantify the number of puncta, mRFP-GFP-LC3B-transfected cells were seeded in a culture plate with 24 wells for 24 h before the treatment. Images were then recorded by fluorescence microscopy. The red puncta that overlay with the green ones and appear yellow in merged images are indicators of autophagosomes, while the free red puncta that do not overlay with the green ones and appear red in merged images are indicative of autolysosomes. The number of puncta per cell was determined using Image-Pro plus 6.0. More than 10 cells were analyzed for each condition.

### Western Blot Analysis

Briefly, whole cell lysates, cytoplasm, and nuclear lysates were prepared with a protein extraction kit (Merck Millipore), and protein concentrations were detected using a Biomate 3s (Thermo Fisher Scientific). First, 5 µg of protein was subjected to electrophoresis on a 10–15% SDS-PAGE gel and then transferred onto a polyvinylidene difluoride membrane (Merck Millipore) by electrophoresis. After blocking for 1 h in 5% BSA, the membranes were incubated with antibodies against proteins or β-actin (standard controls) followed by HRP-conjugated secondary antibodies. The signals were detected using a Pierce ECL-plus substrate (Thermo Fisher Scientific) and scanned with a Fluor Chem FC3 camera system (Protein-Simple, USA). The images were analyzed with the Alpha View software (AIC, USA), and quantitative analyses are presented graphically.

### Statistical Analyses

Statistical analyses were performed using the Graphpad Prism 5 software (GraphPad Software, Inc., USA). The descriptive data are expressed as the mean ± SEM, and numerical data between two groups were compared using the homogeneity of variance or Wilcoxon Mann–Whitney tests, as appropriate. Differences in the mean values of various groups were analyzed using one-way ANOVA with the Tukey–Kramer multiple comparison test or the Kruskal–Wallis *H* test with the Nemenyi test. *P* < 0.05 was considered statistically significant.

## Author Contributions

TZ performed most of the experiments, analyzed the data, and wrote the manuscript. CM designed the project, evaluated and interpreted data, wrote the manuscript, as well as financed and supervised the study. XW, YZ, GY, and DC edited various parts of the manuscript. CX, XM, and SW helped with the experimental design. FW, LB, XL, HL, and QL performed some of the experiments and evaluated the data. YX supervised data analysis and edited the manuscript.

## Conflict of Interest Statement

The authors declare that the research was conducted in the absence of any commercial or financial relationships that could be construed as a potential conflict of interest.
